# Supplementary material for: The Role of Interoceptive Sensitivity and Hypnotizability in Motor Imagery
Source: Brain Sci. 2024 Aug 19;14(8):832. doi: 10.3390/brainsci14080832 (PMC11353101; doi:10.3390/brainsci14080832)
Supplement: Supplementary file 1 [file brainsci-14-00832-s001.zip › brainsci-3107859-supplementary.pdf]

Supplementary Electronic Material

Table S1. Decomposition of alpha PSD region x condition x group interaction.

			F	df	*p*
		**highs**			
R > M			6.51	1,15	.022
R=V=K					
		**mediums**			
R >M			13.27	1,10	.005
R > V			5.87	1,10	.036
R > K			5.42	1,10	.042
**M=V=K**					
		**lows**			
				t	*p*
R		FC < PO		10.80	.0001
M		FC< PO		11.79	.0001
V		FC< PO		12.62	.0001
K		FC< PO		14.62	.0001
	FC	R > M		5.81	.0001
		R =V=K			
	PO	R > M		4.12	.0001
		R >V		2.39	.024
		R > K		2.06	.050
		**M < V**		2.36	.026
		**M < K**		3.10	.005
		V=K			

Table S2. Decomposition of low and high beta PSD effects and interactions.

effect	**low beta**	*F*	df	*p*	
	R >M	91.43	1,50	.0001	
	R > V	38.71	1,50	.0001	
	R > K	37.05	1,50	.0001	
	**M<V**	17.04	1,50	.0001	
	**M<K**	14.92	1,50	.0001	
	V=K				
	**high beta**				
	highs	ns			
	mediums	12.02	1,10	.006	left <right
	lows	ns			
			t	*p*	
	R	FC < PO	11.73	.0001	
	M	FC< PO	12.46	.0001	

V		FC< PO	12.59	.0001
K		FC< PO	13.04	.0001
	FC	R > M	5.58	.0001
		R =V		
		R >K	3.05	.004
		V=K		
		M <V	4.45	.0001
		M < K	3.44	.0001
	PO	R=M		
		R =V		
		R => K		
		M=V		
		M < K	2.45	.018
		V=K		

Appendix S1

Visual imagery

"*Now please imagine doing the same movement you did a few minutes ago. You can clearly see your left arm flexing up to the shoulder while your fingers touch your thumb one by one from the index to the little finger. You can distinctly see your hand approaching your body until your arm touches your shoulder, then your fingers go back touching your thumb from the little finger to the index as your arm extends back to the starting position on the thigh. Please, continue imagining doing this movement ten times at the same pace, with your arm flexing and extending and your fingers touching your thumb from the index to the little finger on the way up, and from the little finger to the index on the way down) and then imagine that movement*".

Kinesthetic imagery

"*Now, please imagine repeating the same movement you did a few minutes ago. You can feel the tension growing in your left biceps as it flexes up to the shoulder, while the muscles in your forearm start contracting and your fingers touch your thumb one by one from the index to the little finger. Notice the increasing extension in your triceps as your hand approaches your body, until the arm contacts your shoulder. Then, as your fingers start going back, feel the contraction in your triceps, while your fingers touch your thumb from the little finger to the index and your arm extends back to the starting position on your thigh. Please continue imagining this movement ten times at the same pace, with your bicep and triceps alternating in contraction and relaxation, and your fingers touching your thumb from the index to the little finger on the way up, and from the little finger to the index on the way down*").
